# Deciphering the selectivity of inhibitor MKC9989 towards residue K907 in IRE1α; a multiscale *in silico* approach

**DOI:** 10.1039/d0ra01895c

**Published:** 2020-05-26

**Authors:** Sayyed Jalil Mahdizadeh, Antonio Carlesso, Leif A. Eriksson

**Affiliations:** Department of Chemistry and Molecular Biology, University of Gothenburg 405 30 Göteborg Sweden leif.eriksson@chem.gu.se +46 317869117

## Abstract

The selectivity of the ligand MKC9989, as inhibitor of the Inositol-Requiring Enzyme 1α (IRE1α) transmembrane kinase/ribonuclease protein, towards the residue K907 in the context of Schiff base formation, has been investigated by employing an array of *in silico* techniques including Multi-Conformation Continuum Electrostatics (MCCE) simulations, Quantum Mechanics/Molecular Mechanics (QM/MM) calculations, covalent docking, and Molecular Dynamics (MD) simulations. According to the MCCE results, K907 displays the lowest p*K*_a_ value among all 23 lysine residues in IRE1α. The MMCE simulations also indicate a critical interaction between K907 and D885 within the hydrophobic pocket which increases significantly at low protein dielectric constants. The QM/MM calculations reveal a spontaneous proton transfer from K907 to D885, consistent with the low p*K*_a_ value of K907. A Potential Energy Surface (PES) scan confirms the lack of energy barrier and transition state associated with this proton transfer reaction. Covalent docking and MD simulations verify that the protein pocket containing K907 can effectively stabilize the inhibitor by strong π–π and hydrogen bonding interactions. In addition, Radial Distribution Function (RDF) analysis shows that the imine group formed in the chemical reaction between MKC9989 and K907 is inaccessible to water molecules and thus the probability of imine hydrolysis is almost zero. The results of the current study explain the high selectivity of the MKC9989 inhibitor towards the K907 residue of IRE1α.

## Abstract 2

The high selectivity of inhibitor MKC9989 towards Lys907 of IRE1α is explained by the unique p*K*_a_ properties of the lysine.

## Graphical abstract

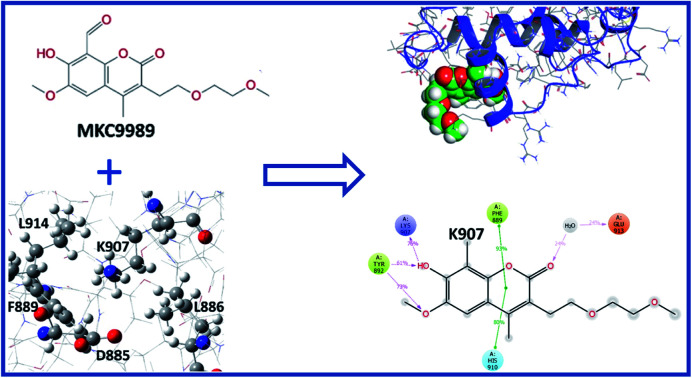

## 1. Introduction

Inositol-Requiring Enzyme 1α (IRE1α, hereafter IRE1) is a transmembrane endoplasmic reticulum (ER) bound bifunctional kinase and endoribonuclease protein crucial for the unfolded protein response (UPR) signaling. Upon ER stress, IRE1 homodimerizes, trans-autophosphorylates and oligomerizes, resulting in endoribonuclease activity with the excision of a 26 nucleotide intron from the X-box binding protein 1 (XBP1) mRNA.^1,2^ The mature form of XBP1s initiates the gene response to ER stress.^3–5^

UPR signaling is a promising target for the treatment of numerous diseases^6^ and targeting IRE1 activity, the most conserved sensor of the UPR, could elucidate the molecular mechanism of these complex signaling pathways. An inhibitor called MKC9989, able to target the RNase domain, has been developed by using a series of *in silico*, *in vitro* and *in vivo* data.^7–9^

**Fig. 1:**
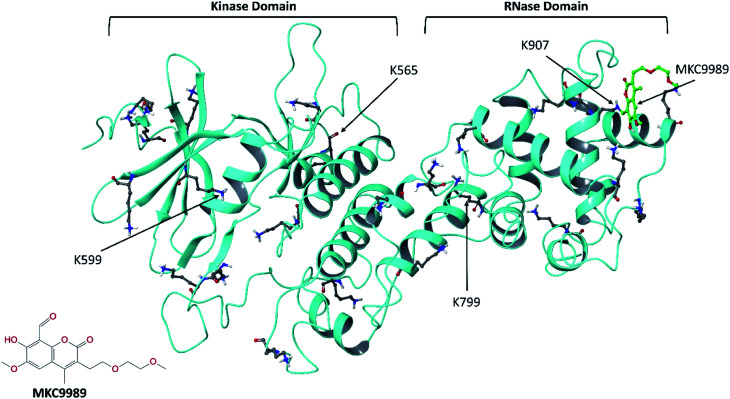
The crystallographic structure of the cytosolic domain of IRE1 (PDB code: 4PL3) with the inhibitor MKC9989 covalently bound to residue K907 in the RNase domain. The protein backbone and all 23 lysine residues are shown in ribbon and ball-and-stick representation, respectively. The inset picture is the 2D molecular structure of MKC9989.

From a functional point of the view, the MKC9989 inhibitor (Fig. 1) belongs to a series of IRE1 RNase inhibitors termed hydroxy aryl aldehydes (HAA) where the aldehyde moiety reacts chemically with the amine side chain of K907 through a Schiff base reaction.^7,9^ The molecular mechanism that can rationalize the high selectivity specifically towards K907 out of the total 23 lysine residues is not yet well-understood. In the current work, complementing our previous study,^10^ we have conducted an in-depth investigation of this phenomenon.

To properly address the high selectivity of MKC9989 towards residue K907, we have considered different factors before and after the Schiff base formation, such as the capability of the lysine residues to be engaged in Schiff base reaction and the stability of the newly formed Schiff base. The most critical prerequisites for Schiff base formation is that the side chain amine group of lysine should be in its uncharged (deprotonated or neutral) state^11^ to be able to nucleophilically attack the carbaldehyde carbonyl group of MKC9989.^7^ Hence, the p*K*_a_ value of the lysine plays a crucial role for its reactivity toward Schiff base formation.^12^ Another important factor is the capability of the receptor pocket environment in stabilizing the newly bound inhibitor.^13^ In this context, water accessibility to the imine group is an important parameter, since these can be easily hydrolyzed in the presence of water through a reverse Schiff base reaction.^14^

p*K*_a_ is a quantity that describes the amount of free energy change of a residue during the protonation process at a given pH.^15^ At neutral pH, the p*K*_a_ of an isolated lysine in water is 10.4,^16^ thus the side chain amine group would be protonated. However, in a protein, the microenvironment properties around each residue can significantly alter its pH dependence towards protonation, mainly by electrostatic interactions, which results in significant shifts in p*K*_a_ values.^17^ It has been demonstrated that a p*K*_a_ shift of just one pH unit may have a large impact on the conformational change and biological activity of biomolecules.^16^ Antosiewicz *et al.*^18^ demonstrated that it is almost impossible to estimate reasonable values of p*K*_a_ for a fully rigid protein, especially with dielectric constants smaller than 20. The explicit motions of side chains can be considered in MD simulation with a predefined protonation state and by averaging the p*K*_a_ values over each structure throughout the trajectory.^19,20^ While this strategy seems efficient, it however ignores the energy difference between configurations in the MD trajectory, which in turn contributes to the free energy change of protonation.^16^

Herein, the Multi-Conformation Continuum Electrostatics (MCCE) approach^19^ was instead employed to calculate p*K*_a_ values of all 23 lysine residues in the cytosolic domain of the IRE1 (PDB/Chain ID: 3P23.A). MCCE combines continuum electrostatics and molecular mechanics.^19^ The protein side chain motions are simulated explicitly through rotamer sampling while the dielectric effect of the solvent and bulk protein is modeled by continuum electrostatics. The electrostatic interactions were computed by means of the Poisson–Boltzmann equation through multiple DelPhi runs as implemented into MCCE v2.7, and the resulting p*K*_a_ values were obtained using Metropolis Monte Carlo sampling of all conformers as a function of pH.^19^

To confirm the MCCE simulation results, Quantum Mechanics/Molecular Mechanics (QM/MM) calculations^21^ were performed to evaluate the explicit interaction between residues around K907 in the IRE1 RNase domain (residues 830–964). Moreover, potential energy surface (PES) scans and hydrogen bond strength calculations were performed using the same approach.

Except for residue K907, for which the IRE1/MKC9989 covalent complex is available (PDB code: 4PL3),^9^ covalent docking^22^ was used in the absence of crystallographic structures to provide covalent IRE1/MKC9989 complexes with a total of four representative lysine residues.^10^ The stability and non-bonded interactions of the covalently bound MKC9989 inhibitor with two buried (K907 and K599) and two solvent accessible (K656 and K799) lysines (Fig. 1) were studied using a series of MD simulations.^10^ Also, the solvent accessibility of the newly formed imine groups, after Schiff base reaction, was quantitatively studied by means of radial distribution function (RDF) of water molecules around the imine groups during the MD simulation.

Upon combining the results of MCCE simulation, QM/MM calculations and MD simulations, the data in full explains the observed high selectivity of MKC9989 and related HAA-based inhibitors towards K907.^10^

## 2. Methods

### 2.1. Protein preparation

The IRE1 apo monomer was built by keeping chain A from the crystal structure (PDB ID: 4PL3)^9^ and removing all other chains, ligands and cofactors, including the covalently bound MKC9989. The residue involved in Schiff base formation, K907, was restored to its original lysine side chain form. The IRE1 apo monomer was then prepared using the protein preparation wizard implemented in the Schrödinger program package.^23^ Hydrogen atoms were incorporated and possible missing side chain atoms and missing loops were added using the Prime program.^11,24^ Tautomeric and protonation states of all residues were determined at pH = 7.4. The prepared structure of IRE1 was then refined using the OPLS3e force field^25^ in a restrained minimization procedure.

### 2.2. MCCE simulation

The MCCE technique^19^ was employed to calculate p*K*_a_ values of all residues in the prepared IRE1 structure. All side chain atoms were allowed to move while the protein backbone was considered to be rigid. Also, all protonatable residues were allowed to be in neutral or ionized states. PARSE radii and charges were used for the protein atoms.^26^ The MCCE simulations were carried out with three different protein dielectric constant values (*ε* = 2, 3, and 4), while the solvent dielectric constant was kept constant at 80 with a salt concentration of 150 mM. It has been demonstrated that a protein dielectric constant of 2 is consistent with PARSE charges^27,28^ and it is also commonly used for many studies based on Molecular Mechanics Poisson–Boltzmann (MM-PB)^29^ or Molecular Mechanics Generalized Born (MM-GB)^30^ methods. The value of *ε* used in a calculation depends on the level of physical detail represented explicitly in a model.^31^ For fully microscopic simulations, *ε* = 1 because all contributions to dielectric relaxation in the protein–water system are treated explicitly.^32^ However, microscopic models of the system with implicit treatment of induced dipoles (electronic polarization) will be best described by using *ε* = 2.^32^ On the other hand, when the both induced dipoles and protein relaxation (nuclear relaxation or reorientation of dipoles) are treated implicitly, *ε* > 4 should be used.^33^ In the MCCE simulation, protein relaxation is simulated explicitly through rotamer sampling while the dielectric effects of the solvent and bulk protein are modeled implicitly by continuum electrostatics. Therefore, according to literature, *ε* = 2 can be an appropriate choice for dielectric constants of the protein.^34^

The torsion energies and van der Waals parameters were evaluated based on the standard AMBER force field.^35^ Herein, we employed FULL MCCE analysis where the positions of all side chains were first determined by (a) rotation around all rotatable bonds by 45° increments followed by (b) pruning rotamers with undesirable clashes. Afterwards, two step optimizations were carried out for dihedral angle optimization and for proton addition followed by final pruning of conformers. A total number of 7936, 7110, and 6467 conformers were considered for DelPhi^36^ pairwise interaction calculations and Monte Carlo sampling procedure at *ε* = 2, 3, and 4, respectively.

### 2.3. MKC9989 ligand preparation

The structure of the MKC9989 ligand was obtained from the co-crystallized PDB structure (4PL3) and its aldehyde moiety was re-modeled as appropriate (Fig. 1). The pre-reactive MKC9989 ligand was then prepared in the Schrödinger LigPrep program at pH = 7.4 using the OPLS3e force field.^25^ Finally, the most stable conformer of the ligand was determined using the MacroModel program implemented in the Schrödinger suite, and used for covalent docking studies. Herein, we used the Mixed Torsional/Low-Mode sampling (MTLMOD) method.^37^ The probability of torsion rotation/molecular translation was set to 0.5 with 100 steps per rotated bond. The minimum and maximum distances for low-mode motions were set to 3.0 and 6.0 Å, respectively. The energy window for saving the structures and maximum atom deviation cutoff were assigned to be 5.0 kcal mol^−1^ and 0.5 Å, respectively. The OPLS3e force field was employed with water as solvent. The extended cutoff option was chosen to truncate the non-covalent interactions (*i.e.* 8.0, 20.0, and 4.0 Å for van der Waals, electrostatic, and hydrogen bond interactions, respectively). The Polak–Ribiere conjugate gradient (PRCG)^38^ method with maximum iteration of 2500 and convergence threshold of 0.05 kJ mol^−1^ Å^−1^ was used as minimization algorithm acting on the gradient.

### 2.4. Covalent docking

Besides the co-crystallized complex with MKC9989 covalently bound to K907, the prepared MKC9989 ligand was covalently docked into IRE1 using CovDock in Schrödinger.^39^ The additional selected lysine residues (K599, K656 and K799) were defined as active residues and the imine condensation reaction between the amine group of the lysine and the reactive aldehyde of MKC9989 was simulated. Energies were calculated using the MM-GBSA rescoring function.^40^ As described in ref. 10, by performing Solvent Accessible Surface Area (SASA) analysis of the twenty-three lysine residues in the IRE1 cytosolic domain, two representative buried lysines (*i.e.* K907 and Lys599) and two solvent exposed lysines (*i.e.* K656 and K799) have been chosen. The calculated SASA values are 6.1, 11.5, 104.0, and 200.0 Å^2^ for K907, K599, K799, and K656, respectively. The general idea was to choose representative lysines in a way that they are at a distance from each other to avoid ligand–ligand interactions and minimize allosteric effects during the IRE1 + 4 MKC9989 MD simulations (Fig. 1).

### 2.5. MD simulation

The highest scoring poses from the covalent docking at each of the three additional lysines were, together with the K907-bound crystallographic structure, subjected to an MD simulation in NPT ensemble using the Desmond MD simulator engine in Schrödinger^41^ employing the OPLS3e force field.^25^ Water molecules were modeled using the TIP3P force field.^42^ Periodic boundary conditions were applied in all directions along with a 10 Å water buffer around the protein in an orthorhombic simulation box. The net charge of the system was balanced using the proper number of counter ions (*i.e.* Cl^−^/Na^+^) and the salt concentration was set to 150 mM to represent physiological conditions. Temperature (300 K) and pressure (1 atm) were controlled using the Nose–Hoover thermostat^43^ and the Martyna–Tobias–Klein barostat,^44^ respectively. The initial minimization and relaxation protocol consisted of (a) NVT Brownian dynamics with restraints on solute heavy atoms at *T* = 10 K for 100 ps, (b) NVT simulation at *T* = 10 K with restraints on solute heavy atoms for 12 ps, (c) NPT MD simulation at *T* = 10 K with restraints on solute heavy atoms for 12 ps, (d) NPT MD simulation at *T* = 300 K with restraints on solute heavy atoms for 12 ps, and (e) NPT MD simulation at *T* = 300 K without restraints for 24 ps. Following equilibration, the MD simulation was then run for 100 ns with a trajectory sampling frequency of 100 ps in the production step.

### 2.6. QM/MM calculations

**Fig. 2:**
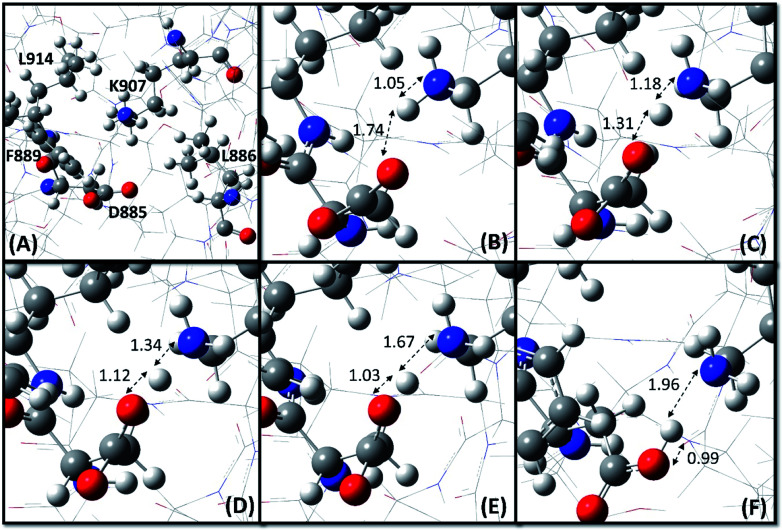
Proton transfer from K907 to D885 during the structure optimization. (A) Initial configuration (step #1), and zoomed in at (B) step #15, (C) step #25, (D) step #35, (E) step #45, and (F) optimized configuration (step #352). The NH and OH bond lengths are in Å. Oxygen, nitrogen, carbon and hydrogen atoms are shown as red, blue, gray and white spheres, respectively.

The ONIOM (Our own N-layered Integrated molecular Orbital and molecular Mechanics) technique,^45^ as implemented in Gaussian 16,^46^ was employed to carry out the QM/MM calculations on the RNase domain of IRE1 (residues 830–964). The residues K907, D885, F889, L886 and L914 (Fig. 2A) were considered as the QM region and treated by the dispersion corrected Minnesota M06-2X functional^47^ along with the 6-31+G(d,p) basis set, while the rest of the RNase domain was assigned as the MM region for which the Universal Force Field (UFF)^48^ was employed. The structure optimization started with ionized states of K907 and D885. The potential energy surface (PES) scan and hydrogen bond strength evaluation were performed at the same level of theory. The PES scan was performed as a function of OH distance (0.7–2.2 Å with step length 0.1 Å) and OHN angle (180 ± 75°).

## 3. Results and discussion

### 3.1. Pre-Schiff base reaction analysis

**Fig. 3:**
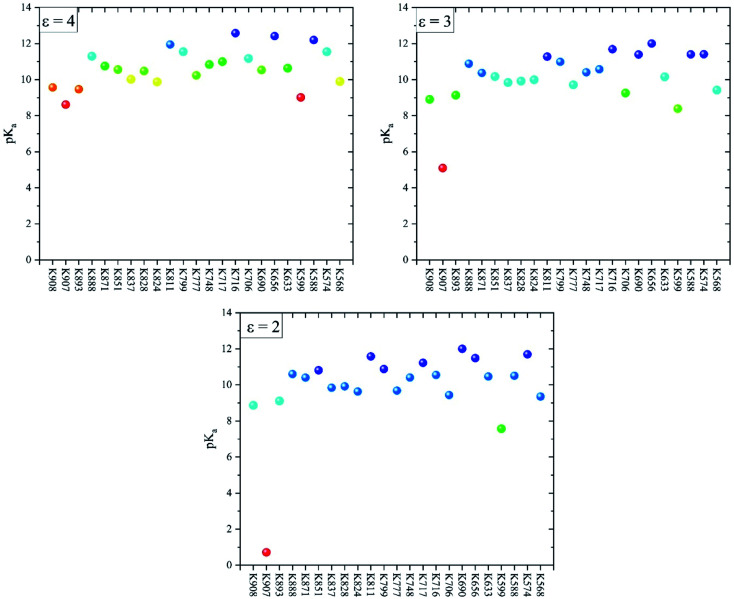
The calculated p*K*_a_ values of all 23 Lys residues of the IRE1 cytosolic domain at three different protein dielectric constants (*i.e.* ε = 4, 3, and 2). Intermediate residues colored relative to the lowest (red) and highest (blue) value in each set.

Fig. 3 shows the p*K*_a_ values of all lysine residues extracted from the MCCE simulations at protein dielectric constants 2, 3, and 4. The lowest p*K*_a_ values belong to K907 and K559 with values 8.6, 5.1, and 0.7 (K907) and 9.0, 8.4, and 7.6 (K559) at *ε* = 4, 3, and 2, respectively. The p*K*_a_ values of these two buried residues decrease as *ε* decreases which indicates their preferences to be deprotonated at lower protein dielectric constants. It is surprising that p*K*_a_ of K907 at *ε* = 2 is totally acidic compared to the other lysine residues (Fig. 3). The p*K*_a_ values of the remaining lysines are essentially unaffected by the change in dielectric constant, and range between 8 and 12.5. A closer look at the p*K*_a_ values of the other residues around K907 (Fig. 2A) indicates that D885 exerts an equally abnormal increase in p*K*_a_ with decreasing protein dielectric constant. The p*K*_a_ values of D885 are calculated to be 1.8, 3.5, and 9.6 at *ε* = 4, 3, and 2, respectively. This result clearly illustrates an interaction between K907 and D885 inside the hydrophobic cavity, which is significantly enhanced as the protein dielectric constant is reduced.

It has been demonstrated that the p*K*_a_ of a buried ionizable residue is extremely sensitive to the protein conformation and polarity of the surrounding microenvironment.^17^ In internal polar hydrophilic microenvironments, the p*K*_a_ values are close to those of individual amino acids in water. However, in apolar hydrophobic microenvironments, the p*K*_a_ of ionizable residues will shift making the neutral state more favorable.^17^ Isom *et al.* have studied the effect of the polarity of the microenvironment on the p*K*_a_ of lysine residues by engineering 25 variants of staphylococcal nuclease with lysine residues at internal positions.^17^ They found a very large reduction in p*K*_a_ of some lysine residues, as low as 5.3, inside a hydrophobic pocket in close proximity to carboxylic group containing residues.

To confirm the MCCE results and to investigate the interaction between K907 and the surrounding residues, a series of QM/MM calculation were conducted based on dispersion corrected DFT using the M06-2X functional. We started the structure optimization process with K907 and D885 in their respective ionized states, based on the prepared pdb structure (PDB code: 4PL3). As Fig. 2 shows, a clear proton transfer occurs from K907 to D885 during the optimization. This implies that K907 prefers to remain deprotonated inside the highly hydrophobic pocket surrounded by F889, L886, and L914. The transferred proton maintains a hydrogen bond between the donor and acceptor atoms in the optimized structure, with OH, NH, and ON distances 0.99, 1.96, and 2.95 Å, respectively, and OHN angle 171.8°. Additional calculations revealed that this hydrogen bond is relatively weak, with an energy of 10.1 kcal mol^−1^.

**Fig. 4:**
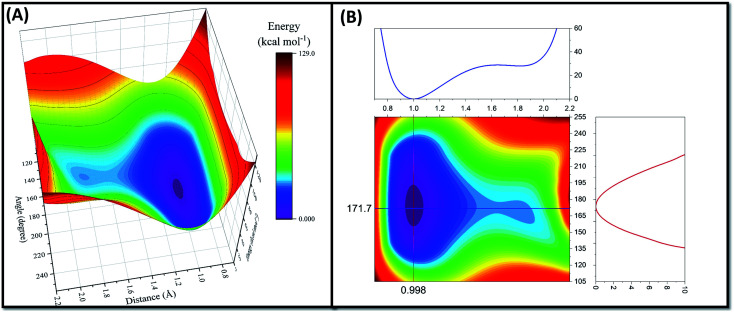
The PES (kcal mol^−1^) along the proton transfer pathway from K907 to D885. (A) 3D PES as a function of OH distance (Å) and OHN angle (degrees). (B) Contour map profile of PES with optimum values of OH bond and OHN angle depicted with straight blue and red lines, respectively.

Since the proton transfer has occurred during the structure optimization process, it is expected that there is no or very low energy barrier for this reaction mechanism. Indeed, a PES scan, performed as a function of OH distance (0.7–2.2 Å with step length 0.1 Å) and OHN angle (180 ± 75°), confirms the hypothesis (Fig. 4). As seen in Fig. 4A, the PES along the proton transfer pathway possesses a double minimum profile feature with a very shallow well around the amine nitrogen atom of K907 residue. Indeed, the energy barrier for proton transfer from K907 to D885 (0.82 kcal mol^−1^) is 35 times lower than the proton transfer in the opposite direction (28.89 kcal mol^−1^). This very small energy barrier could not prohibit the spontaneous proton transfer during the optimization as it is close to the RT value at 298 K (∼0.6 kcal mol^−1^); *i.e.* the proton will easily pass through this minimal barrier just by means of thermal motion. Two main phenomena explain how proton transfer can occur during the minimization: (1) strong electrostatic interaction of positively charged K907 and negatively charged D885 leads to an enhancement in zero-point vibrational energy and amplitude of N–H vibration, which consequently can overcome the small energy barrier. (2) It is well-known that, due to light weight of the proton, quantum effects cannot be neglected for reactions involving proton transfer.^49^ Proton tunneling is a quantum mechanical phenomenon that can justify how protons can transfer from one microstate to another one by passing through the energy barrier between the two.^49^

The contour map profile of the PES (Fig. 4B) shows that the optimum values for OH distance and OHN angle are 0.998 Å and 171.7° which are consistent with the structural parameters of the optimized configuration. The results of the p*K*_a_ calculations from the MCCE simulations and the spontaneous proton transfer phenomenon from the QM/MM calculations provide a clear picture linking the prerequisites for Schiff base chemical reaction to the high selectivity of MKC9989 towards K907.

### 3.2. Post-Schiff base reaction analysis

**Fig. 5:**
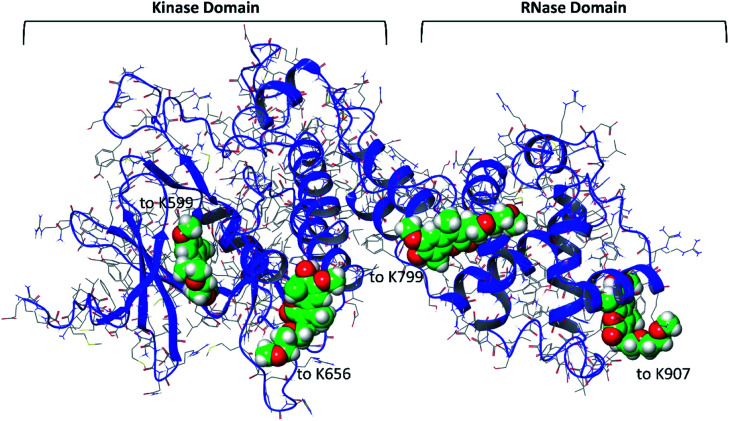
The structure of the four covalently bound MKC9989 molecules (space filling model) to the four selected lysine residues of the cytosolic part of IRE1.

To investigate the parameters which may influence the stability of the newly formed Schiff base between four representative lysine residues^10^ and the aldehyde moiety of MKC9989, the covalent complexes between MKC9989 and K599 (kinase pocket), K656 and K799 (solvent exposed), and K907 (RNase site), were explored further (Fig. 5). The docking score values and MM-GBSA free energy of binding (Δ*G*_b_) (Table 1) indicate that the pocket containing K907 is the favored site for covalently bound MKC9989 to IRE1. However, given the difficulty of the CovDock program to reproduce the exact crystallographic pose of MKC9989 towards K907,^50^ we decided to use the structure from PDB ID 4PL3 at this particular site in the further investigations.

**Table 1:** Docking score and MM-GBSA free energy of binding (Δ*G*_b_) of MKC9989 to the different sites of IRE1

Docking site	Docking score	MM-GBSA Δ*G*_b_ (kcal mol^−1^)
K599	−4.61	−38.04
K656	−3.18	Not reported^a^
K799	−3.73	Not reported^a^
K907	−5.90	−61.12

a Not reported by CovDock program.

**Fig. 6:**
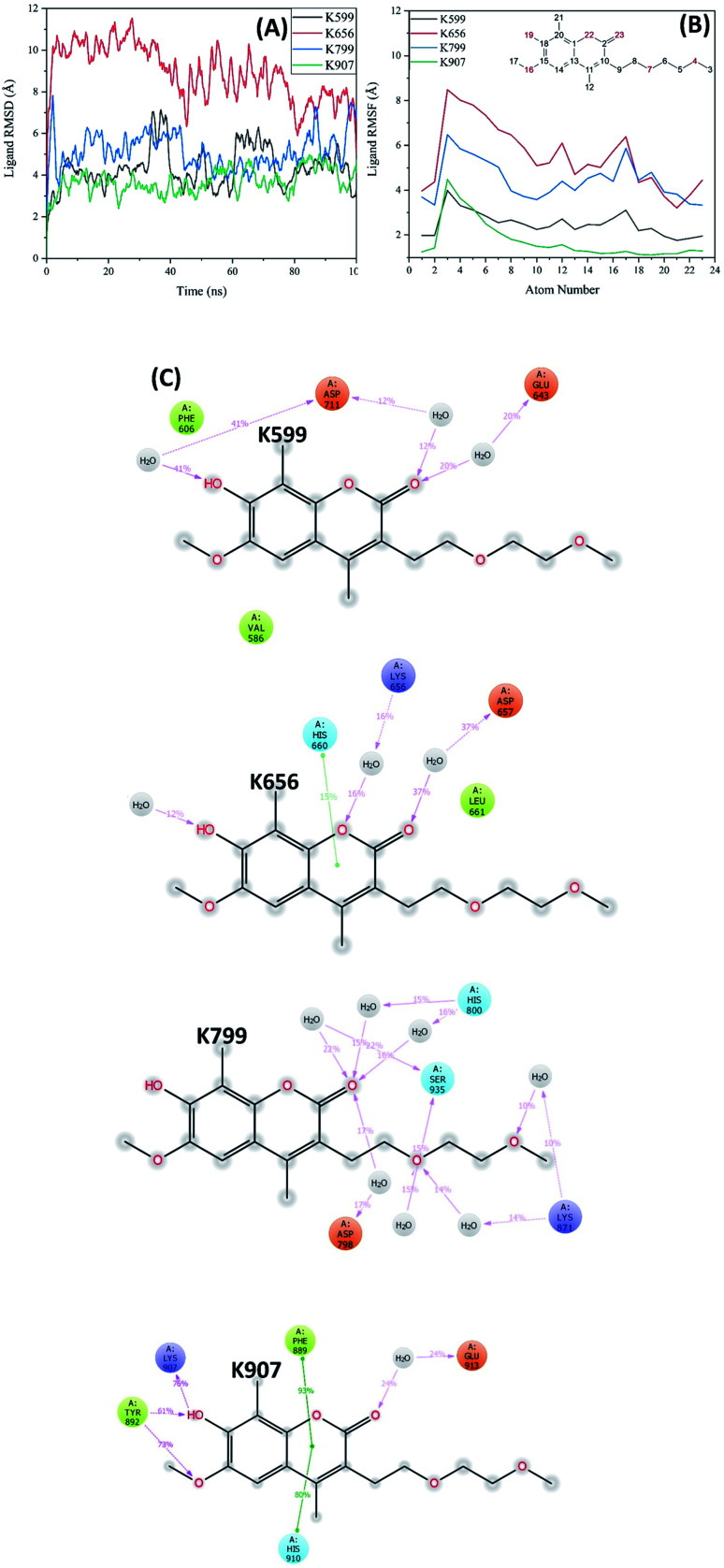
(A) RMSD of MKC9989 ligands covalently attached to the different lysine residues during the 100 ns MD simulation. (B) RMSF plot of MKC9989 ligands covalently bound to the different lysine residues during the MD simulation. The atom numbers are according to the 2D structure of the ligand shown in the inset panel. (C) Protein–ligand interaction diagrams for MKC9989 attached to the different lysine residues. Abundance of interactions (% of MD simulation trajectory snapshots), and solvent exposure of the ligand atoms (grey spheres) are also displayed.

The IRE1/4×MKC9989 complex was then subjected to a 100 ns MD simulation for further evaluation of ligand dynamics and solvent accessibility of the formed imine bond. As mentioned, solvent accessibility is an important parameter since the imine group can easily be hydrolyzed in the presence of water molecules in a reverse Schiff base reaction. Fig. 6A shows the RMSD of the covalently attached MKC9989 ligands during the 100 ns molecular dynamics simulation. As this figure indicates, the ligand movement inside the RNase pocket (K907) is smaller compared to the MKC9989 ligands covalently bound to K599, K656 and K799.

Fig. 6B shows the RMSF plot of the ligand bound to the four different lysines. Ligand RMSF plots describe the ligand fluctuations broken down to individual atoms. The atom numbers are according to the 2D structure of the ligand shown in the inset panel of Fig. 6B. The ligand RMSF will give us better insight on how ligand fragments interact with the protein and their entropic role during the binding event. As the figure indicates, the RMSF values of the ligand bound to K907 are considerably lower than those attached to the other lysines (specifically solvent exposed ones). The differences in RMSF values are significant for atoms around the covalent bonding position (*i.e.* two fused rings) which confirms the stability of MKC9989 bound to K907 within the RNase domain.

**Fig. 7:**
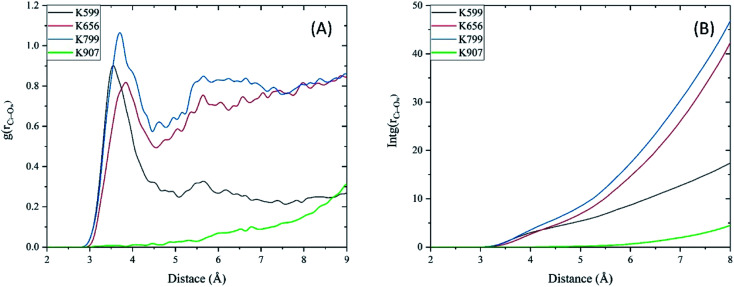
(A) RDF of water molecules, and (B) average number of water molecules (integral of RDF) around each imine group as a function of distance between the carbon atom of the imine (C_i_) and oxygen atoms of water (O_w_) during the MD simulations.

The protein–ligand interaction diagram (Fig. 6C) shows that MKC9989 inside the pocket containing K907 exhibits π–π interactions with F889 and H910 during 90% and 83% of the simulation time, respectively. The very low solvent accessibility of the imine group formed within this region (Fig. 7) can be associated to these π–π interactions that prevent water molecules from diffusing into the pocket and make, along with the strong hydrogen bonds to Y892 and K907, the ligand molecule more rigidly bound inside this pocket in comparison with the other sites (Fig. 6B). Common for MKC9989 bound to any of the other three lysines are water-assisted hydrogen bonds to the interacting residues, and a high degree of solvent exposure.

In Fig. 7A we present the radial distribution function, RDF (g(r)), of water molecules around each imine groups as a function of distance between the carbon atom of the imine (C_i_) and oxygen atoms of water (O_w_) during the MD simulation. The lack of the first peak in the RDF plot of MKC9989 inside the pocket containing K907 shows that the corresponding imine group is fully inaccessible to water molecules, which prevents imine hydrolysis process from taking place. Fig. 7B shows the integral of RDF during the MD simulation which quantitatively represents the average number of water molecules around each imine groups, as a function of distance. As Fig. 7B indicates, as opposed to the other three lysines, there are no water molecules around the imine group formed during the reaction between MKC9989 and K907, up to 6 Å away from the C_i_ atom. As discussed earlier, the strong π–π interactions between MKC9989 and F889 and H910 may block the water molecules from diffusing into the pocket region.

## 4. Conclusions

Herein, the high selectivity of the UPR inhibitor MKC9989 towards K907 in the IRE1 RNase pocket is investigated by means of multiscale *in silico* techniques including MCCE simulations, QM/MM calculations, covalent docking, and MD simulations. The MCCE simulations showed that the lowest p*K*_a_ values belong to K907 and K559 with values of 8.6 and 9.0, 5.1 and 8.4, and 0.7 and 7.6 at *ε* = 4, 3, and 2, respectively. The p*K*_a_ values of in particular K907 decreases dramatically as *ε* decreases, which indicates preferred deprotonation at lower protein dielectric constants. The MCCE simulations also identified a critical interaction between K907 and D885 within the hydrophobic pocket, which was markedly enhanced at lower protein dielectric constants. The QM/MM calculations showed a spontaneous proton transfer from K907 to D885, in agreement with the low p*K*_a_ value of the K907 residue. The PES scan confirmed the negligible energy barrier (0.82 kcal mol^−1^) associated with this proton transfer reaction. The results from covalent docking and MD simulations demonstrated that the pocket containing K907 can effectively stabilize the inhibitor's structure by strong π–π and hydrogen bonding interactions. RDF analyses furthermore confirmed that the imine group formed in the chemical reaction between MKC9989 and K907 is totally inaccessible for water molecules, and thus the probability of imine hydrolysis is negligible.

These results give new insights into the selectivity of the MKC9989 inhibitor towards K907 out of the 23 lysine residues of IRE1, and provide a suitable starting point for QM/MM or QM/MM-MD studies of the MKC9989-IRE1 reaction. This work also shows how different computational tools can be utilized to describe the local factors leading to Schiff base formation in biological systems.

## Authors contributions

All authors conceived the study. S. J. M. performed the computations, analyzed the data and wrote the first draft. All authors revised the text.

## Data availability

All simulation protocols, input files for QM/MM and MCCE calculations, and output structures form covalent docking are provided as tarballs (.tar.gz) freely accessible at zenodo.org as DOI: 10.5281/zenodo.3621358.

## Conflicts of interest

The authors declare no conflicting interests.

## Supplementary Material
